# Exploring the views of patients' and their family about patient‐initiated follow‐up in head and neck cancer: A mixed methods study

**DOI:** 10.1111/ecc.13641

**Published:** 2022-07-04

**Authors:** Ava Lorenc, Colin Greaves, Joan Duda, Jo Brett, Lauren Matheson, Tessa Fulton‐Lieuw, Denis Secher, Pat Rhodes, Gozde Ozakinci, Paul Nankivell, Hisham Mehanna, Marcus Jepson, PETNECK2 Research Team

**Affiliations:** ^1^ QuinteT Research Group, Population Health Sciences, Bristol Medical School University of Bristol Bristol UK; ^2^ School of Sport, Exercise and Rehabilitation Sciences University of Birmingham Birmingham UK; ^3^ Supportive Cancer Care Research Group, Faculty of Health and Life Sciences Oxford Brookes University Oxford UK; ^4^ Institute of Head and Neck Studies and Education (InHANSE), Institute of Cancer and Genomic Sciences University of Birmingham Birmingham UK; ^5^ Patient representatives; ^6^ Division of Psychology, Faculty of Natural Sciences University of Stirling Stirling UK; ^7^ University Hospitals Birmingham NHS Foundation Trust Birmingham UK

**Keywords:** head and neck cancer, patient education, patient information, psychological, supportive care, users

## Abstract

**Objective:**

The objective of this work was to explore head and neck cancer (HNC) patients' and their family members' views on acceptability and feasibility of patient‐initiated follow‐up (PIFU), including concerns and anticipated benefits.

**Methods:**

Patients were recruited from UK HNC clinics, support groups and advocacy groups. They completed a survey (*n* = 144) and/or qualitative interview (*n* = 30), three with a family member. Qualitative data were analysed thematically, quantitative data using descriptive statistics.

**Results:**

Preference for follow‐up care in HNC was complex and individual. Many patients thought PIFU could beneficially reallocate health care resources and encourage self‐management. Patients' main concerns with PIFU were losing the reassurance of regular clinic appointments and addressing mental well‐being needs within PIFU, possibly using peer support. Patients were concerned about their ability to detect recurrence due to lack of expertise and information. They emphasised the importance of a reliable, direct and easy urgent appointment service and of feeling supported and heard by clinicians. Patients believed family and friends need support.

**Conclusion:**

PIFU may be feasible and acceptable for certain HNC patients, providing it addresses support for mental well‐being, provides quick, reliable and direct clinician access and information on “red flag” symptoms, and ensures patients and their caregivers feel supported.

## 1 BACKGROUND

There are 12,200 new head and neck cancer (HNC) cases in the United Kingdom every year (Cancer Research UK, n.d.). HNC has a high patient burden due to problems with speech, voice, swallowing, pain and disfigurement. UK HNC guidelines recommend that patients have follow‐up appointments every 2 months for the first 2 years after treatment, and then every 3–6 months for the next 3 years (Simo et al., 2016).

Although UK HNC patients are positive overall about follow‐up care (Wells, Cunningham, et al., 2015), HNC follow‐up often does not meet patients' survivorship and psychosocial support needs (Breen et al., 2017; Lewis et al., 2009; Simcock & Simo, 2016; Wells, Semple, & Lane, 2015). Moreover, current appointment‐based follow‐up protocols may not be the most effective method of cancer recurrence detection (Hall et al., 2019; INTEGRATE (UK ENT Trainee Research Network) et al., 2021; Pagh et al., 2016; Szturz et al., 2020; Zatterstrom et al., 2014) and are resource‐intensive and potentially unsustainable given increasing HNC incidence in the UK (Cancer Research UK, n.d.). Less intensive follow‐up may be appropriate, especially for stage I cancers (Kanatas et al., 2014). New follow‐up paradigms therefore need to be considered (De Felice et al., 2021; INTEGRATE (UK ENT Trainee Research Network) et al., 2021) and researched (Hall et al., 2019; Mueller et al., 2019; Schwartz et al., 2003). Patient‐initiated follow‐up (PIFU) is one potential alternative approach (Whear et al., 2013). The PETNECK2 research programme with an embedded RCT was designed to develop and evaluate an intervention promoting PIFU for HNC.

Patients may prefer PIFU to regular scheduled follow‐up in various conditions (Whear et al., 2013) including endometrial (Beaver et al., 2020; Kumarakulasingam et al., 2019), colorectal (Batehup et al., 2017; Burton et al., 2017) and prostate (Frankland et al., 2019) cancer. PIFU‐based approaches in breast cancer were well received by patients and did not affect quality of life, anxiety or depression (Brown et al., 2002; Chapman et al., 2009; Koinberg et al., 2004; Riis et al., 2020), time to recurrence or death (Koinberg et al., 2004). In HNC, PIFU alone has not been evaluated ‐ studies added elements of PIFU (education/information on seeking help) to current appointment‐based HNC follow‐up, potentially improving appointment compliance (De Zoysa et al., 2017), self‐examination (Vaishampayan et al., 2017) and well‐being (Turner et al., 2019). Preliminary data suggest HNC patients can effectively use patient‐initiated approaches alongside current appointment‐based follow‐up (Boysen et al., 2016; Brandstorp‐Boesen et al., 2019; De Zoysa et al., 2017; Salander et al., 2016).

In a UK survey presenting hypothetical follow‐up scenarios, most HNC patients felt follow‐up was too frequent and preferred less intensive follow‐up with options for reporting problems and requesting appointments, ideally with a nurse (Trinidade et al., 2012). However, another UK survey found only 3% of HNC patients felt follow‐up was too frequent and 83% would prefer regular appointments to PIFU ( Flanagan et al., 2011). Studies of PIFU in other cancers (Brandenbarg et al., 2017; Frew et al., 2010) and countries (Alders & Hermens, 2017; Meregaglia et al., 2017; Mueller et al., 2019) found most patients preferred more intensive health care professional contact, disliked losing the reassurance of regular clinician follow‐up (Brennan et al., 2019; Brown et al., 2002) and were concerned about accessing clinical and psychological support (Chapman et al., 2009). Preference for regular follow‐up may reflect satisfaction with previously received care and an unwillingness to change. Some of HNC patients' perceived barriers to self‐management may also apply to PIFU, including emotional barriers (e.g., fear of recurrence), symptom‐related barriers (e.g., voice problems), structural barriers (e.g., access to health services) and self‐evaluative barriers (e.g., interpersonal self‐evaluative concerns) (Dunne et al., 2018). Facilitators for PIFU in other cancers include convenience (reduced travel/time/cost) (Beaver et al., 2020; Brown et al., 2002; Kumarakulasingam et al., 2019), confidence in quick and easy clinician contact (Beaver et al., 2020; Koinberg et al., 2002) and confidence in recognising symptoms (Beaver et al., 2020; Koinberg et al., 2002).

Given the mixed evidence on PIFU in HNC instead of regular follow‐up, the PETNECK2 study (PETNECK2 STUDY TEAM, 2022) will determine the efficacy of PIFU versus regular scheduled follow‐up for HNC, after imaging at trial entry (12 months after completing treatment) to select patients at low risk of recurrence. PIFU includes rapid access to urgent clinical appointments within 2 weeks, an allied health professional (AHP)‐/nurse‐led patient education session, and an information and support resource (app, website or paper booklet) with information on important symptoms, concerns, patient/caregiver support, living well, and peer support groups, a symptom diary and clinical team contact details.

Due to the novel nature of PIFU in HNC, preliminary research with clinicians and patients was conducted on feasibility, barriers and concerns regarding PIFU (Kieft et al., 2017). Data on clinicians' perspectives are reported elsewhere (Lorenc et al., 2021); this paper reports HNC patients' and family members' views. Pre‐RCT qualitative work can be invaluable in informing study design and development and implementation of new interventions (Husbands et al., 2019; Rooshenas et al., 2016).

## 2 METHODS

The COREQ checklist (Tong et al., 2007) was used in reporting.

### 2.1 Participants

This study consisted of a survey and qualitative interviews with HNC survivors and, for interviews only, their family members (interviewed with patients or separately). Time from treatment was <3 years for interviewees, unspecified for survey participants. All eligible and willing patients participated.

Patients were recruited by participating hospitals, usually by local PI or research nurse at routine clinic appointments or by phone, and through HNC support and patient advocacy groups.

### 2.2 Survey questionnaire

The survey questionnaire (see Appendix S1) was designed to elicit barriers to and enablers of PIFU guided by the COM‐B model (Michie et al., 2011): *c* *apability* (e.g., knowledge; ability to accurately detect symptoms), *opportunity* (e.g., clear channels of communication for reporting symptoms) and *motivation* (e.g., understanding benefits of early intervention/the risks of delay; belief in treatment efficacy; fear).

The PETNECK2 Study Patient Advisory Group (PAG) reviewed patient‐facing information, helped to design the interview topic guides and provided advice on recruitment, and some members participated in the survey. Two PAG members attended programme meetings, contributed to interpretations of findings and reviewed this paper.

Participants completed the survey online or were given/posted a paper copy. The online version included a video explanation by the PAG lead. The survey data were analysed using simple descriptive statistics and simple categorisation of open‐ended responses.

### 2.3 Patient interviews

Interviews were 30–60 min, either online via video‐calling (Microsoft Teams) or phone, by AL (female) or MJ (male) (whom participants did not know beforehand—they knew AL and MJ were non‐clinical staff, researching patient views for PETNECK2 study), audio‐recorded and transcribed verbatim. Notes recorded nonverbal behaviour to supplement/clarify transcripts. Family members were interviewed concurrently or in separate interviews, depending on patient preference. Interview questions covered current follow‐up and views about PIFU, including acceptability, barriers and perceived benefits (see Appendix S2).

Interview data were analysed using thematic analysis (Braun & Clarke, 2006), including elements of codebook and reflexive thematic analysis (Braun & Clarke, 2019), with no participant input. Codes were drawn both from the interview questions and (inductively) from the data, and synthesised using familiarisation; coding; generating and developing themes (Braun & Clarke, 2019). Data were examined for credibility, context, language, negative cases and rival explanations (Patton, 1999). Reflexive themes not from questions or prompts are emphasised in the results. AL analysed interviews, with MJ commenting on codes and importance of themes and subthemes. AL and MJ are senior qualitative researchers with extensive health care research experience but limited knowledge of HNC and no clinical qualifications or background. Nvivo software (version 20.5.1.940) was used to facilitate data analysis.

The study was approved by North East ‐ Tyne & Wear South Research Ethics Committee, the Health Research Authority and Health and Care Research Wales, reference 20/NE/0102. All interview participants received study information and provided written consent prior to interview, and transcripts were pseudonymised. The survey was completed anonymously by participants unless they provided their email address.

## 3 RESULTS

### 3.1 Participants

Thirty‐nine patients expressed an interest in the interviews, three were not eligible (more than 3 years post‐treatment), four did not respond to emails, one had health issues and one declined. Of the remaining 30, three had a family member join them in the interview (one partner, one spouse and one daughter). A total of 144 patients completed the survey. Fifteen patients took part in both the survey and an interview (most did the survey first). Participant demographics are presented in Table 1.

**TABLE 1:** Participants' demographic characteristics

	Survey (*n* = 144)	Interviews (*n* = 30)
**Age (mean)**	64 years	Not collected
**Gender**		
Male	85 (59%)	17 (57%)
Female	55 (38%)	13 (43%)
No data	4	
**Education**		
School up to 15/16 years old	67 (47%)	13 (43%)
School up to 18 years old	31 (22%)	
College/other	0	5 (17%)
Undergraduate university	16 (11%)	8 (27%)
Postgraduate	25 (17%)	3 (10%)
No data	5	1
**Ethnic group**		
White	Not collected	29 (97%)
Bangladeshi		1
**Time since completion of treatment**		
Average (mean)	40 months	23 months
Minimum	0 (treatment ongoing)	1 month
Maximum	20.8 years	38 months

Over half of both survey and interview participants were male, which is slightly lower than the general HNC population (69%) (Cancer Research UK, n.d.). Almost a third had a university education, which is comparable to the UK population (Office for National Statistics, 2011) but likely higher than the HNC population (Conway et al., 2010). Interviewees were less ethnically diverse (97% White) than UK mouth cancer cases (78%) (National Cancer Intelligence Network, 2009).

Results are presented around six key interview themes, with survey data integrated into themes to add detail. Qualitative quotes from interviews and open‐ended survey responses are presented in Tables 2, 3, 4.

**TABLE 2:** Quotes exemplifying the themes

Theme	Subthemes	Examples
Mixed preference for follow‐up		*I'd have welcomed something like that [PIFU] if it had been offered to me probably around about nine months [post‐treatment] (P39, interview)* *I wouldn't mind either one or the other if I'm honest (P63, interview)*
Anxiety about losing the reassurance of regular follow‐up	Reassurance of regular follow‐up	*he always puts the camera up my nose and he has a good look round in there, and it puts my mind at rest that it's not malignantly crawling away in there (P67, interview)*
	Losing that reassurance	*I don't like the sound of it [PIFU] …. I think I need the reassurance of actually being checked, somebody actually looking at him (75, family member of patient 66, interview)* *I think that's [PIFU] a good idea, as such, but I think it is nice to have the reassurance [of regular appointments] (P46, interview)*
	Especially for anxious patients	*I know a lot of people‐ I mean I've got a guy who, we went through treatment together, it [PIFU] would horrify [him], the fact that if he can't get seen every three months, as he's due, because he's an anxious person (P49, interview)*
The need for mental health support and peer support	Importance of mental well‐being support	*I think anxiety plays a major role in cancer patients and anything that may relieve this would be great (P46, survey)*
	Peer support for mental well‐being	*[when I am] talking in head and neck survivors' groups, not only is there usually some useful advice (or at least an ‘Oh yeah, me too’) but also quite often one will find someone worse off. Not pleasing, of course, but makes one realise that perhaps it's not all bad (P14, survey)* *[the] best support I have found [is] from people who have shared the cancer experience, they are the only ones who truly understand (P58, survey)*

**TABLE 3:** Quotes on themes of confidence seeking help, benefits of PIFU and concerns about detecting symptoms

The importance of confidence in seeking help	Confidence in seeking help currently.	*I've always been incredibly impressed with the openness of the team I was involved in and the ability to access them at period of times when you felt a little bit stressed by the situation (P39, interview)* *thankfully I have been able to contact my cancer clinic when I have had concerns about symptom changes and the appropriate action has always been taken. That in itself builds personal confidence (P17, survey)*
	Need for reliable access to clinicians	*I think the single most important thing really is … the access to care if they are worried (P45, interview)* *Central number to call that is answered immediately and does [not] keep callers waiting or uses voice response that asks patients to select “Option this, for …” which sometimes lead to a dead end, forcing the caller to call again. This can be quite frustrating to a patient who is already stressed out (P49, survey)* *To get an appointment, if necessary, to see someone quickly will also help manage their [patients'] anxiety & stress levels. Would like to know they [clinicians] are there should I need them, especially in the first 6–12 months post treatment (P105, survey)* *Reassurance of a ‘fast track’ access route to specialist advice and support—allocated keyworker/designated named professional (P94, survey)*
	Feeling supported	*[important for patients to know] that it's still okay to ring and ask “silly” questions (P59, survey)* *[I] know that any concerns I have will be taken seriously (P94, survey)* *I have a good rapport with my consultant and clinical nurse specialist, so would have no problem contacting them (P31, survey)* *It would be useful for concerned patients to have a clear idea of how long before someone actions their concerns (P22, survey)*
Benefits of PIFU	Prioritising health care resources	*I'm in total agreement that you could actually save a lot of resource this way (P83)* *anything to keep cost down of cancer recurrence, or need for professional intervention, is worthwhile. My goal is to be of as little bother as possible (survey)*
	Self‐management	*[with PIFU] you're being trusted to be involved, for a start … You're taken seriously as a person (P83, interview)* *[recording symptoms] is a self‐monitoring process which may provide some reassurance (P57, survey)* *the weight loss, prior to getting diagnosed, I just ignored it. And I think, if I hadn't have mentioned to the dentist*—*it was just a passing thing about my ulcer in the back of the throat*—*I would have just carried on and not bothered about my health. But because of what's happened, I'm now more aware (P73, interview)*
	Altruism	*whether or not it [taking part in RCT] was beneficial to me, in the future it could be particularly helpful to others that have to undergo similar types of treatment (P68, interview)* *I want to help and I want to try and find ways to help people in the future …. [that's] why I'm trying to do stuff like this [taking part] (P31, interview)*
Concern about detecting symptoms	Lack of expertise	*I don't know if I'm meant to be looking for anything else. I feel round my neck in case … If I start feeling not right, but I can't, I don't know what I'm really looking for, apart from a lump (P70, interview)* *whether the change is just due to old age, you know, or whether it's … not necessarily cancer that it's due to …. And I think it's, it's, you know, getting that sorted out is not an easy thing to do (P37, interview)* *I would hate to put the onus on me to check correctly (survey)*
	Lack of information	*It would be good if there was a list of symptoms to look out for and a time frame for how long you should go before getting in touch. The reason I think this would help is that when I had earache I was scared to get in touch in case it was something and nothing and I did not want to bother anyone (P21, survey)*
	Self‐examination upsetting	*And I look [at] my tongue and mouth, but when I look at it, it makes me sad …. Interviewer: Because it doesn't look the same, and so‐?* *Respondent: No, no. Especially as you can see the cut [from surgery] every day in the morning when you get up … when I look [for signs of recurrence], sometimes I do cry, you know? Interviewer: Yeah. So that would put you off looking, maybe, would it? Respondent: Yes (P87, interview)*

**TABLE 4:** Quotes about family and friends

Optional involvement	*My husband was very supportive … But he's not very scientific. And if you try to involve him in something like that [PIFU], I think he'd pull away … Unless people [family members] are really in there and want to be part of a team (P45, interview)*
Need for openness	*The key issue is being able to talk about your (the patient) worries/concerns—this is true for the patient AND their family & friends (P105, survey)* *Ask. Basic, I know, but “I didn't like to ask/be a bother” kills people (P104, survey)*
Importance of family and friend support	*My wife has been amazing and I am sure other spouses are the same (P57, survey)*
Support for family and friends	*Haha. So funny. Nothing [currently provided for family and friends] at all of course—the NHS treats cancer. It doesn't see the need to support patients or caregivers. Sadly they treat the disease, not the patient and not the whole family (P22, survey)* *If they are helping the patient to check for recurrence signs, again, they need to know WHAT to look for (all signs/symptoms) & who to contact if they find something of concern (P105, survey)*

### 3.2 Attitudes to follow‐up and PIFU

During interviews, interviewees expressed their preference for type of follow‐up. Around a third preferred existing clinic follow‐up, a third PIFU, and a third did not have a preference. Although definitive associations cannot be made, interviewees further from treatment were generally keener on PIFU and felt it was only appropriate from 1‐year post‐treatment. There was no apparent difference in preference according to education level or HNC type/severity. Some cited the convenience of not attending follow‐up appointments as a benefit to PIFU, especially during the Covid‐19 pandemic. Interviewees welcomed nurse delivery of PIFU—as long as nurses were HNC experts—some felt they were more approachable than consultants. For some survey respondents, consistency of contact person was important. Only one interviewee disagreed, preferring a consultant (but appreciated that this may cost more).

Although most interviewees welcomed a digital tool, proficiency with or access to technology was a concern and a barrier to using an app to record symptoms for 14 (10%) survey respondents. Interviewees recommended non‐digital options due to perceived wide‐ranging capabilities and preferences among HNC patients, and most survey respondents wanted options (digital/paper).

Interviewees cited concerns about those less engaged in their health care, anxious patients, older patients and those who lived alone.

### 3.3 Anxiety about losing the reassurance of regular follow‐up and the need for mental health support, including peer support.

Interviewees' most frequently mentioned benefit of current follow‐up (clinical follow‐up every 3‐6 months for 5 years) was the reassurance of thorough checks for recurrence. Of particular importance was clinicians' objectivity and expertise, providing a “safety net,” and seeing someone face‐to‐face. This all contributed to (patients' and family members') feelings of well‐being, support, safety and peace of mind. Loss of reassurance in PIFU was interviewees' main/only concern, even for those supportive of PIFU, and restricted enthusiasm for PIFU. Interviewees were particularly concerned about patients with existing anxiety.

Fear of recurrence was common in interviews, but often only “at the back of my mind,” and many perceived the risk as low, with some intentionally optimistic and unconcerned. There was widespread understanding that recurrence risk reduced over time. Interviewees' fear of recurrence was triggered by a history of cancer (themselves or family) and limited confidence in ability to detect recurrence. Survey data strongly endorsed inclusion of mental well‐being support (anxiety/stress/low mood) within PIFU (77% of respondents), with support “essential” and “invaluable,” with little existing health care support (56% had none).

**FIGURE 1:**
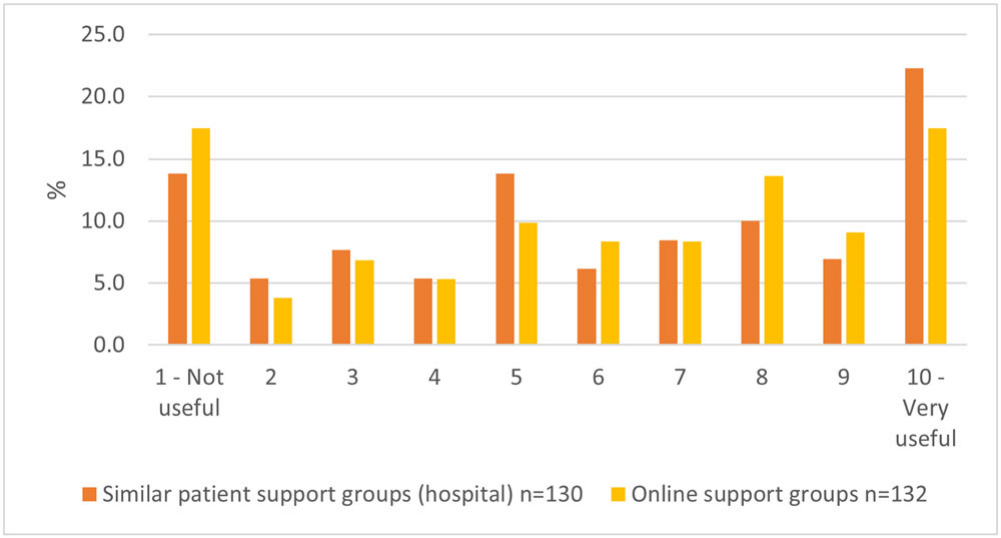
Usefulness of peer support as part of PIFU

From qualitative survey data, the most common mental well‐being support suggestion was peer support (see Figure 1). Interviewees described peer support as talking to others “in the same boat” and emphasised its particular importance in HNC as a rarer cancer. Other mental well‐being suggestions included trustworthy information about cancer and prognosis, general advice to “talk,” seeing a psychologist/counsellor, and contact with clinical team. Some interviewees suggested, without prompting, that PIFU include some regular appointments (e.g., annually), to provide reassurance (“a happy medium”) and PIFU plus appointments was by far the most popular option in the survey.

### 3.4 The importance of confidence in seeking help

**FIGURE 2:**
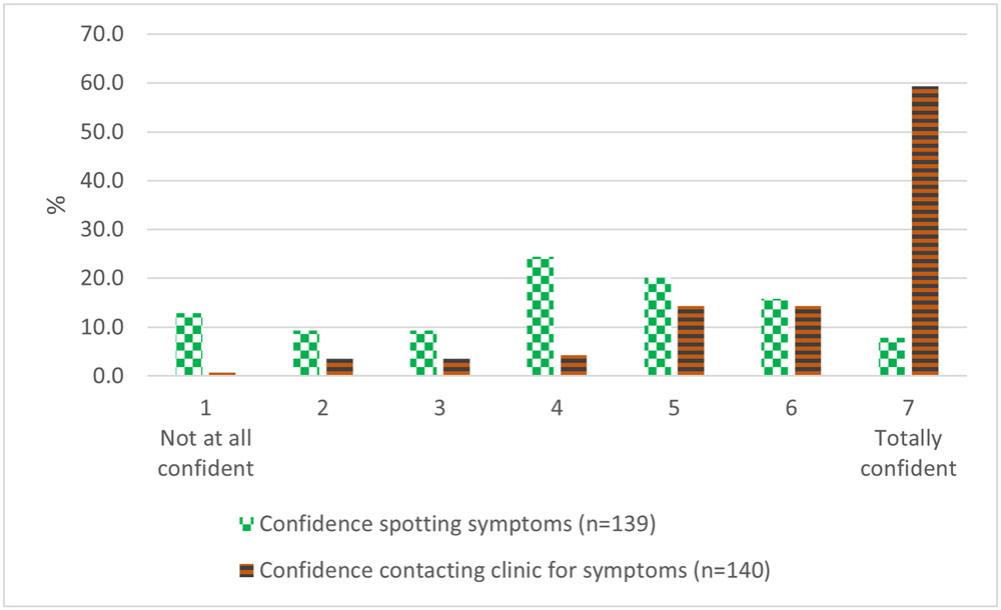
Survey respondents' confidence identifying symptoms and seeking help

Most interviewees and 58% of survey respondents were confident contacting the clinic during follow‐up upon symptom identification (see Figure 2). Most patients (survey and interviews) knew who to contact—mostly consultants, oncologists or surgeons, but 24% of survey respondents would contact GP, 11% nurse, 8% a charity‐funded nurse and 9% another clinician.

Despite confidence, there was some concern about anticipated access to and response from clinicians during PIFU, and 47% of survey respondents anticipated barriers getting appointments. Also, 72% of survey respondents still wanted more information on who to contact and how, and confirmation they would be listened to. Quick, direct, reliable and easy access to a clinician and expert advice was important for interviewees and survey respondents in PIFU, for example, not being put on hold or access via receptionists.

The patient–clinician relationship during PIFU was important, including being taken seriously and appropriate, timely responses (survey respondents), and feeling supported (interviewees).

### 3.5 Benefits of PIFU: prioritising health care resources and shifting responsibility to patients

Many interviewees and a few survey respondents perceived, unprompted, that PIFU could beneficially reallocate health care resources by releasing clinician time or diverting funding to patients in greater need.

Some interviewees saw PIFU as patient‐led care and encouraging self‐care/responsibility, important in cancer recovery and recurrence detection. Survey respondents specifically mentioned using a symptom “diary” as useful for self‐management. Many interviewees emphasised the importance of self‐examination during follow‐up, based on having delayed help‐seeking prior to their original diagnosis.

Many interviewees were altruistically motivated to help the health care system/patients by taking part in clinical trials such as PETNECK2, with some saying they would consider participating.

### 3.6 Concern about detecting symptoms due to lack of information and expertise

Although most survey respondents reported self‐examining—most commonly once a week or more (39%) or once or twice a month (22%)—patients' lack of information was a recurrent concern, including on symptoms (only 7.8% were “totally confident” re symptoms to look for—see Figure 2), how to examine (important for 70% in the survey) and who to contact, as described above; 42% of survey respondents welcomed a video or demonstration of symptom‐checking. A couple of interviewees cautioned against symptom information overload, which might alarm patients.

Lack of information and expertise created apprehension among interviewees about recurrence symptom detection, in particular distinction from the multitude of changes due to cancer, treatment or age. The difficulty of seeing mouth and throat symptoms and subtlety of original symptoms were concerning, especially if cancer had not originally been suspected. One interviewee found self‐examination extremely upsetting as a reminder of the cancer and its impact.

### 3.7 Support of family and friends is important in PIFU.

Interviewees' views on involvement of family and friends in PIFU, including helping with symptom‐checking, were mixed. Most suggested it is non‐compulsory, as family members or friends may be unable or unwilling to be involved.

Some survey respondents cited barriers to family and friends' involvement, including living alone or not wanting to burden them. Some advised openness between patients and family members/friends around mental well‐being and informing and involving family and friends about the cancer and how they can help. Support for family and friends as part of PIFU was strongly endorsed by survey respondents (104/139 [75%]), with many (39%) saying there is currently no support.

## 4 DISCUSSION

Interviewees had mixed preferences for regular follow‐up or PIFU, with PIFU possibly more acceptable as time since treatment increased. Although the option of a digital information resource was welcomed, technology access and competence were important barriers, with non‐digital options also required. Patients' most common concern about PIFU was losing the reassurance of regularly seeing experts and being examined for recurrence, particularly for patients perceived as anxious. Mental well‐being support was therefore important within PIFU, perhaps via peer support. Most patients were confident seeking help but wanted the urgent appointment service within PIFU to be reliable, direct and easy, and emphasised needing to feel supported and listened to. Most patients recognised the importance of and performed self‐examination but were concerned about missing symptoms of recurrence due to lack of information and expertise and physical barriers to self‐examination. Despite these concerns, patients mentioned several advantages of PIFU, including beneficial reallocation of health care resources and opportunities for self‐management and quicker recurrence detection, to help themselves and the health care system/other patients. Views on family and friend involvement in PIFU were mixed, but support for family and friends was strongly endorsed due to their important role and current lack of support. This study has identified specific implications for PIFU pathways—see Table 5.

**TABLE 5:** Implications for PIFU design

Component	Details
Format	Delivered by expert nurses
	Variety of format options
	Emphasise self‐care
Clinician access	Easy and reliable access to urgent appointments
	Encourage patients to contact hospital directly rather than via other providers, e.g. GPs
	Family and friend direct access to clinicians
	Training for all clinic staff in PIFU
Family and friends	Optional family and friend involvement
	Signposting to support for family and friends
Self‐examination	Address physical and emotional barriers to self‐examination
Patient information	Reliable information from health care system
	Symptoms to be alert to, particularly anything beyond ‘lumps’, subtle symptoms, e.g., a ‘feeling’, and outside the HNC area
	How to distinguish between long‐term effects, treatment side effects or age‐related changes and signs of recurrence
Mental health support	Consider including (optional) peer support
	Anticipate concerns and fear of recurrence and signpost to sources of support

Follow‐up visit frequency and content is contentious in HNC (Szturz et al., 2020). Our results suggest follow‐up preference is complex and individual—indeed, one of the advantages of PIFU is it allows a more flexible and patient‐centred approach to follow‐up that current follow up strategies do not. Previous HNC studies identify patient preference for both regular follow‐up (Flanagan et al., 2011; Mueller et al., 2019) and PIFU (Alders & Hermens, 2017; Brennan et al., 2019; Kumarakulasingam et al., 2019; Trinidade et al., 2012). Barriers and enablers to PIFU identified are consistent with COM‐B categories of capability, opportunity or motivation (Michie et al., 2011) (as our questionnaire was informed by the COM‐B model). Qualitative data did not identify any major additional influences apart from the notion that barriers and enablers (such as anxiety or preference) vary over time, dynamic variation important to consider in PIFU design. Participants likely overemphasised barriers to PIFU as regular follow‐up users—in practice if PIFU became a standard of care, patients would understand and expect a change in follow up over time from the start of their treatment, and a service using PIFU will have additional capacity due to fewer scheduled appointments. However, PIFU should perhaps be considered as one of a range of follow‐up options, in line with recommendations and patient preference (Gasson et al., 2014) for nuanced, flexible follow‐up tailored to individual (Brennan et al., 2019; INTEGRATE (UK ENT Trainee Research Network) et al., 2021; Wells, Cunningham, et al., 2015) and local (Lester & Wight, 2009) needs, for example, planned risk‐stratified follow up in a UK HNC clinic (De Felice et al., 2021). Cancer stage is also likely to be an important consideration, with PIFU more appropriate for early cancer (Kanatas et al., 2014).

Fear of recurrence is a major concern among HNC patients (Rogers et al., 2020), and regular follow‐up provides the reassurance of regular appointments, expert specialists and rapid access to tests (Brandenbarg et al., 2017; Lewis et al., 2009). Although PIFU may diminish cancer patients' reassurance (Brown et al., 2002; Kumarakulasingam et al., 2019; Lewis et al., 2009) and increase fear of recurrence (Jeppesen et al., 2018), conflicting evidence suggests fear of recurrence (Sheppard et al., 2009) and concerns are similar during PIFU and regular follow‐up (Chapman et al., 2009). Also, the reassurance of regular follow‐up is often temporary, with anxiety returning by the next appointment (Lewis et al., 2009). PIFU may ameliorate this periodic heightened anxiety and its convenience may compensate for lost reassurance (Brown et al., 2002; Kumarakulasingam et al., 2019; Lewis et al., 2009). It seems important to ensure patients on PIFU feel they are being listened to, that their concerns are addressed and that they are not being abandoned. Training for all staff involved, including receptionists and nursing staff, is important. Peer support, including support groups and buddy systems, is common in HNC (Wells, Semple, & Lane, 2015) and may improve emotional well‐being (Egestad, 2013), quality of life (Vakharia et al., 2007) and anxiety and depression (Pateman et al., 2015) in HNC.

Open access health care has been shown to provide a “safety net,” reducing anxiety (Moore et al., 2021), something lacking in our results. This may be as HNC patients delay help seeking, often until their next appointment (Agrawal et al., 2004), despite interventions to encourage contact between appointments, due to not wanting to waste clinicians' time (Kumarakulasingam et al., 2019). It may also reflect participants' concerns about the reliability of the urgent appointment system. Previous research supports our findings that patients, including those on patient‐initiated approaches, appear confident in seeking help for worrying symptoms, including knowing how (Chapman et al., 2009; Whitehead et al., 2019) and when (Gasson et al., 2014) to make contact, but still have concerns about urgent access to specialists and appointments (Beaver et al., 2020; Koinberg et al., 2002; Lewis et al., 2009). PIFU may increase help‐seeking confidence (Beaver et al., 2020) and education, information and encouragement are important (De Zoysa et al., 2017), as well as patients feeling secure in the patient‐clinician relationship and seeing clinicians as knowledgeable and trusted (Koinberg et al., 2002).

The anticipated financial benefits of PIFU for the health care system may reflect patients' concerns that regular follow‐up appointments waste health professionals' time (Beaver et al., 2020; Kumarakulasingam et al., 2019), their satisfaction with health care (3, 31) and public understanding of funding problems faced by the UK national health service (52).

Patients may find PIFU empowering (Moore et al., 2021), as suggested by some interviewees. Self‐management is valued and advocated by patients with endometrial cancer (Beaver et al., 2020; Kumarakulasingam et al., 2019) and has benefits in HNC (Dunne et al., 2019a), although it is hampered by the challenges patients face during follow‐up (Dunne et al., 2019b). Interventions need to support patients to develop self‐management (Dunne et al., 2019b) and health literacy skills (Clarke et al., 2021), perhaps using holistic needs assessments, common in UK HNC follow‐up (Wells, Semple, & Lane, 2015). As Beaver et al state, there is a distinction between “no follow‐up, relying on patient contact” and “supported self‐management” (Beaver et al., 2020). Implementation of PIFU needs to focus on patient understanding of what PIFU is and does (Beaver et al., 2020) and emphasise empowering aspects of PIFU and the improved ease and timeliness of access to clinicians, with reassurance of support.

Patient education and information on recognising signs of recurrence is important (De Zoysa et al., 2017; Gasson et al., 2014; Koinberg et al., 2002; Zatterstrom et al., 2014) and appears to be currently limited. In other cancers, barriers to self‐examination include fear of recurrence, and facilitators are knowledge and confidence (Brown et al., 2002; Dieng et al., 2019; Koinberg et al., 2002; Muktar et al., 2015), but it is not known if these apply to HNC. Our results suggest lack of patient information and expertise, practical barriers, inability to distinguish from other changes and finding self‐examination upsetting may be important, but more research is needed.

Lack of support for family and friends is recognised in HNC and results in caregiver burden (Hanly et al., 2016). In other cancers caregivers' unmet needs include fears about cancer, disease‐related information and emotional support for themselves (Sklenarova et al., 2015).

### 4.1 Strengths, limitations and future research

Data collection was informed by strong patient involvement, and combining interviews and survey responses provided rich, detailed data, including many detailed responses to survey open‐ended questions.

The key limitations were the data collection tools and possible sample biases. Data are also limited by the lack of clinical information on participants, in particular their staging or risk of recurrence, and more research is needed to explore the influence of these factors on feasibility of PIFU. The questionnaire was bespoke (based on a published format) (Michie et al., 2011), which may introduce bias from measurement error/limited validity or reliability, although responses were well distributed with no apparent floor or ceiling effects. Conducting interviews virtually/by phone (necessitated by Covid‐19 pandemic restrictions) limited interpersonal interaction but did allow for a wide geographical spread of participants. The sample may not represent all HNC patients, as many of the interviewees were regular clinic attendees and many were involved in other research—other patients may be less confident seeking help or engaging with PIFU. Preferences for follow‐up are likely to differ for patients who have not yet experienced regular follow‐up. The sample characteristics suggest that the findings may not generalise to ethnic minority patients or those with lower educational levels, and more research is needed to establish this.

Family member/friend recruitment was limited and, given the emergent theme around their importance as supporters and the importance of supporting them, further research is desirable, especially superficially during follow‐up 1 year post‐treatment.

Researchers conducting the interviews were not clinically qualified or experienced in HNC, which may have resulted in more or less probing questions due to naivety. Naivety also may have introduced misunderstandings or meant more nuanced themes were missed, although interpretations were reviewed and discussed with clinically experienced co‐authors.

This study has informed the PETNECK2 research programme, including the design of the intervention and the RCT, and has highlighted the need for the programme to consider which patients PIFU is suitable for. This study has identified other areas for future research in HNC, including the use of/potential for peer support and self‐management, barriers and facilitators to self‐examination and the role of family and friends and their support needs.

## 5 CONCLUSION

Preference for follow‐up care in HNC is complex and individual and it is unlikely that PIFU will be suitable for all patients. Patients' main concern with PIFU is the loss of reassurance, and so this, along with possible effects on mental well‐being of removing regular check‐ups, needs to be addressed, possibly through peer support. However, technologically competent patients without major anxiety or fear of recurrence may well be willing to try PIFU and this could potentially benefit both health services and patients. It is important that the urgent appointment service within PIFU is reliable, direct and easy, that patients feel supported and heard, that PIFU provides clear information on self‐examination, and that family and friends are also supported.

## CONFLICT OF INTEREST

None of the authors have a conflict of interest to declare.

## AUTHOR CONTRIBUTIONS

AL: literature searching, data collection and analysis, manuscript preparation. CG: data collection and analysis, manuscript preparation. JD: data collection and analysis, manuscript preparation. JB: study design, manuscript editing. LM: study design, manuscript editing. TFL: study design, recruitment, manuscript editing. DS: patient representative input into study design, data collection and analysis and manuscript editing. PR: patient representative input into study design, data collection and analysis and manuscript editing. GO: study design, manuscript editing. PN: guarantor of integrity of entire study, study design, manuscript editing. HM: guarantor of integrity of entire study, study design, manuscript editing. MJ: qualitative study design, data collection and analysis, manuscript editing.

## Supporting information


**Appendix S1:** Survey questionnaire
**Appendix S2:** Interview topic guide

**Appendix S3.** PETNECK2 Research Team

## Data Availability

Author elects to not share data.
