# Supplementary material for: Exploring the views of patients' and their family about patient‐initiated follow‐up in head and neck cancer: A mixed methods study
Source: Eur J Cancer Care (Engl). 2022 Jul 4;31(6):e13641. doi: 10.1111/ecc.13641 (PMC9787693; doi:10.1111/ecc.13641)
Supplement: Supplementary file 1 — Appendix S1: Survey questionnaire Appendix S2: Interview topic guide [file ECC-31-e13641-s002.docx]

# Appendix 1: Survey questionnaire

**HELPING US TO DEVELOP OUR PATIENT-SUPPORT PROGRAMME**

**Patient-initiated** **surveillance versus clinical follow-up in head and neck cancer**

(the PETNECK 2 study)

*“This study is funded by the National Institute for Health Research (NIHR) under its Programme Grants for Applied Research Programme (project reference NIHR200861). The views expressed are those of the author(s) and not necessarily those of the NIHR or the Department of Health and Social Care.”*

Currently, patients with head and neck cancer attend a clinic every 2-6 months for 5 years to see how they are getting on and check if the cancer has come back (known as a recurrence). We know from previous research that it is more likely for a recurrence of the cancer to be found by a patient who notices a change in their body or in their symptoms, rather than in a routine clinic appointment with the doctor We are planning to develop a new way to follow-up head and neck cancer patients who have a low risk of recurrence. Instead of bringing patients to hospital every few months routinely, patients would phone for an appointment when they think they need one.

We need your help in developing this new system. The aim of this questionnaire is to seek your views about what we should include in the new follow-up system and how best to present it to patients For further information about our research study, please also refer to the Patient Information Sheet.

**NOTE: By completing the questionnaire, you are providing agreement to participate in the questionnaire study. Please do not include any additional personal details (e.g. names or addresses) in any of the free text boxes. Thank you!**

1. About you

| **Age** |  | | |
| --- | --- | --- | --- |
| **Gender** |  |  | Male  Female  Other gender identity, please specify: ______________  Prefer not to say |
|  |  |  |  |
|  |  |  |  |
|  |  |  |  |
| **Education Level**  Highest level reached | \|  \| \| --- \| \|  \| \|  \| \|  \| \|  \|   Secondary school up to 15/16 years  Secondary school up to 18 years  Additional training (e.g. vocational or trades)  Undergraduate university Postgraduate university | | |
| **Who do you live with?** | \|  \| \| --- \| \|  \| \|  \|   I live alone  I live with a partner, or spouse  I live with other adults or children | | |
| **When was your last head or neck cancer treatment? (i.e. Month / Year)** |  | | |
| **How often do you currently attend the follow-up clinic at the hospital?** |  | | |
| **What kind of checks do you have when you attend?** |  | | |

**2. Aims of the new follow-up system**

Listed below are a number of things we think need to be included in the new follow-up system. These have come from previous discussions with patients with head and neck cancer, their families, doctors and nurses. Please tell us what you think about each one.

**2.1 Checking for symptoms:** We plan to ask people who have a low risk of cancer recurrence to check once or twice a month for possible signs that the cancer might be coming back. For instance, this could include changes in your voice, pain in your head/neck lasting several weeks, or a new lump in the head or neck area.

How often do you (or someone else) currently check for possible signs that your cancer may be coming back?

| **Never** | **Less than once a month** | **Once or twice a month** | **Once a week** | **Twice a week, or more** |
| --- | --- | --- | --- | --- |

What signs or symptoms do you check for? (Please list.)

Please say **who** does this (for example yourself, someone you are close to, or both)?

How confident are you that you know what symptoms to look for?

| **Not at all confident** |  |  |  |  |  | **Totally confident** |
| --- | --- | --- | --- | --- | --- | --- |
| 1 | 2 | 3 | 4 | 5 | 6 | 7 |

**Who** would you ask if you thought a symptom might be a sign of your cancer coming back?

What is the best way to engage family and friends to help to check for symptoms?

Quick questionnaire: If we asked you to check every few weeks for possible symptoms, which of the following might help you to do this?

Please tick all statements that apply to you …

| *I would have to ….* |  |  | *If any of these apply to you, please feel free to tell us more* |
| --- | --- | --- | --- |
| Know more about what to do | e.g. knowing what symptoms to look for or how to check them |  |  |
| Build my confidence about checking | e.g. feel confident that I am doing it right |  |  |
| Overcome some physical problems | e.g. get around any physical difficulties like limited range of movement in head/neck |  |  |
| Overcome obstacles in my mind | e.g. overcome anxiety about getting it wrong or about finding out something I don’t want to know |  |  |
| Get help from someone else | e.g. ask a friend or member of my family to help do the checks |  |  |
| Learn by seeing | e.g. watch someone else do it |  |  |
|  |  |  |  |
| Be prompted to do it | e.g. receive a text reminder when I need to do it |  |  |
| Get a person to remind me | e.g. a friend or family member |  |  |
|  |  |  |  |
| Feel that it is important | e.g. have a better understanding of the benefits of detecting possible symptoms early |  |  |
| Feel that it will do me some good | e.g. know that if my cancer did come back there is a good chance that there |  |  |
|  | are treatments that will stop it if it is caught early |  |  |
| Have a clear plan in place | e.g. have an app or a written document for which symptoms I need to monitor and when |  |  |
|  |  | |  |
| *Anything else?* | *Please specify what other support you would need or like to have to help you check your symptoms.* | |  |
|  |  | |  |

**2.2 Recording your symptoms:** We may ask patients to make a note of their symptoms every now and then using an “app” (a programme on your mobile phone, or on a tablet or computer). This would involve downloading an app, pressing a few buttons and ticking some boxes /options. You might also be asked to record a sentence to check for changes in your voice. You could be prompted to do this via a reminder on your app. This should all take around 2 minutes. On completion, you would be given some feedback (e.g. “all clear, no worries” or advice to contact the doctor if that is needed).

Do you think this should be part of the new follow-up system?

Yes No

*If yes, why? If no, why not?*

Would you be confident to do this using your own mobile phone, tablet or computer?

Yes No

If not, why not?

Would you be confident to do this using a paper and pen /in a diary?

Yes No

*If not, why not?*

What other options for recording your symptoms might work better?

How often do you think you would be willing to make a record of your symptoms? (Using either a diary or a mobile phone, tablet or computer)

| **Never** | **Less than once a month** | **Once or twice a month** | **Once a week** | **Twice a week, or more** |
| --- | --- | --- | --- | --- |

**2.3 Contacting the cancer specialist team if you spot any symptoms you are worried about.**  This sounds simple, but we know that this is not always easy to do.

How confident are you that you would promptly contact the cancer clinic at your local hospital if you spot a symptom or sign that you may want to discuss?

| **Not at all confident** |  |  |  |  |  | **Totally confident** |
| --- | --- | --- | --- | --- | --- | --- |
| 1 | 2 | 3 | 4 | 5 | 6 | 7 |

When it comes to you contacting the cancer clinic about possible symptoms, we would like to find out more about what would help you to do this.

Please tick all of the following statements that apply to you …

| **To feel comfortable contacting the cancer clinic, I would have to** | | **…** | *If any of these apply to you, please feel free to tell us more* |
| --- | --- | --- | --- |
| Build my confidence | e.g. feel confident that I know what to say or what questions to ask |  |  |
| Overcome some physical problems | e.g. get around any difficulties with hearing or speaking |  |  |
| Overcome some obstacles in my mind | e.g. overcome anxiety about ‘being a nuisance’ or about finding out something I don’t want to know |  |  |
| Get help from someone else | e.g. ask a friend or member of my family to help me make the call, or come with me to the appointment |  |  |
|  | |  |  |
| *I would have to ….* | |  |  |
| Feel that it is important | e.g. have a better understanding of the benefits of reporting symptoms early |  |  |
| Feel that it will  do me some good | e.g. know that if my cancer did come back there is a good chance that there are treatments that will stop it |  |  |
| Feel that I will be listened to? | e.g. know the person I contact and know they will take notice of what I say |  |  |
|  |  | |  |
| *I would have to …* |  |  |  |
| Know exactly what to do | e.g. knowing exactly who to contact and how |  |  |
| Know how to get around any  barriers that the NHS might throw  up | e.g. the receptionist might tell me there are no appointments, or none for some time |  |  |
|  |  |  |  |
| *Anything else?* | *Please specify what other support you would need or like to have to help you to contact the cancer clinic.* | |  |
|  |  | |  |

Do you have any other comments or concerns regarding contacting your cancer clinic if you spotted a symptom or sign that might need further treatment?

**2.4 Support for managing any stress, anxiety or low mood caused by concerns about the cancer returning:** We may try to offer ideas and strategies for managing anxiety or low mood. This might involve breathing exercise or meditations designed to help people to be calm, or linking people to existing information, support services or online programmes to help to manage stress, anxiety or low mood.

Should this be part of the programme?

Yes No Don’t Know

*Why – or why not?*

What advice on managing stress, anxiety or low mood have you been given by your local cancer care team, your GP or other health professionals?

What is the best way to engage family and friends to help to manage stress?

Please add any additional comments or suggestions that you may have on supporting people who have survived a cancer diagnosis to manage stress, anxiety or low mood. What kind of support might help people in this situation to cope?

**2.5 Support for family and friends** (providing support for family and friends who are involved in helping people who have finished treatment for head and neck cancer to monitor their condition at home)

Should this be part of the programme?

Yes No Don’t Know

*Why – or why not?*

If yes, what kind of support should be included, and how should this support be provided?

What support or advice for family and friends have you been given by your local cancer care team, your GP or other health professionals?

What are the most important things that friends and family need to know?

Please add any additional comments that you may have on supporting family and friends to help you to monitor your condition at home.

**2.6 Other possible components**

Are there any possibly useful ideas for supporting checking for symptoms of recurrence and followup which we have missed above?

Yes No

If yes, please tell us what else we could do:

Overall, what are the most important things that patients who have finished treatment for head or neck cancer need to know? What messages do you believe would be useful in helping them to monitor their condition?

***3. Delivering the new follow-up support service***

At the moment, based on our discussion so far with patients, families, and health professionals, we believe that the follow-up service we are planning to develop should include all of the following:

1. *An initial consultation between patient and a member of the cancer care team to assess the patient’s needs and concerns and make a plan of action. This might include checking regularly for any worrying symptoms and seeking help promptly if needed.*
2. *Information for patients which explains some basics of looking after yourself after head or neck cancer and support to manage the after effects and live as normal a life as possible.*
3. *Information about what potentially worrying symptoms to look for, how to check for symptoms, and some symptom-tracking tools.*
4. *Patient-initiated follow-up contact, tailored to meet the needs of each individual. This means that patients would phone for an appointment when they think they need one rather than receive an appointment by post as directed by the hospital.*

A number of possible delivery formats are listed below. We have previously discussed these with experts and patients.

**Please pick the number option to indicate ‘how likely to work’ each option would be for you** in the table below.

| **Format** | **On a scale of 1 to 10, how likely is this to work for you?** |
| --- | --- |
|  | 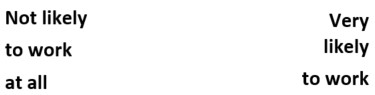 |
| Digital advice and planning support via mobile phone app, tablet or computer. No other support. | 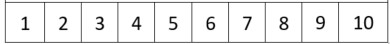 |
| Paper based advice and planning support (e.g. leaflet / workbook). No other support. | 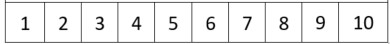 |
| Digital or paper-based support plus 3 or 4 face to face meetings per year with a cancer care specialist (e.g. cancer nurse). | 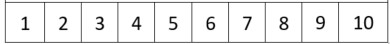 |
| Digital or paper-based support plus 1 face to face meeting and 2-3 phone calls. | 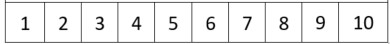 |
| Digital or paper based support plus 2-3 phone calls | 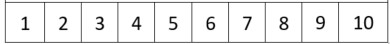 |
| As well as the above options, we may be able to offer the following options. On a scale of 1 to 10, how useful would these additional options be to you? | |
|  | Not useful Very useful |
| Support from groups of similar patients meeting at the hospital | 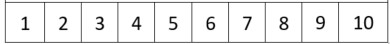 |
| Online support groups | 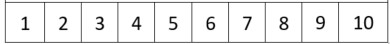 |

OPTIONAL

Finally, would you be willing to take part in a group discussion (a “workshop”) with the research team to help us to develop and start to test our intervention?

 Yes  No

If ‘yes’, please provide us with an email address in the box below so we may contact you:

|  |
| --- |

***Thank you for taking the time to complete this questionnaire.***

***Your responses are greatly valued.***

# Appendix 2: Interview topic guide

Interviews asked about medical history and current follow-up experience and thoughts about cancer recurrence, followed by an explanation of PIFU, and questions about acceptability, feasibility, barriers and perceived benefits, and any concerns regarding the RCT design. Questions covered:

Background

1. Demographics
2. Cancer diagnosis
3. Cancer treatment
4. Follow-up care
5. Self-examination

Views on recurrence

1. Understanding of recurrence
2. Feelings about recurrence
3. Strategies to deal with thoughts of recurrence
4. When contact would be made

Description of PIFU and the PETNeck2 RCT provided to interviewee

Views on the RCT

1. Views on randomisation
2. Potential participation in the RCT
3. Barriers to recruitment and suggested solutions
4. Views on data collection

Views on interventions

1. Feelings about being allocated to PIFU, including nurse education session, how to spot symptoms, role of friends and family, suggestions.
2. Feelings about being allocated to standard care
